# The TMEM106B T186S coding variant increases neurite arborization and synaptic density in primary hippocampal neurons

**DOI:** 10.3389/fnins.2023.1275959

**Published:** 2023-10-13

**Authors:** Quynh Nguyen, Caleb A. Wood, Peter J. Kim, Joanna L. Jankowsky

**Affiliations:** ^1^Departments of Neuroscience, Baylor College of Medicine, Houston, TX, United States; ^2^Neurology, Neurosurgery, and Molecular and Cellular Biology, Huffington Center on Aging, Baylor College of Medicine, Houston, TX, United States

**Keywords:** TMEM106B, T185S, coding variant, neuron development, primary hippocampal neuron, knock-out mouse, knock-in mouse, synapse

## Abstract

The lysosomal protein TMEM106B was identified as a risk modifier of multiple dementias including frontotemporal dementia and Alzheimer’s disease. The gene comes in two major haplotypes, one associated with disease risk, and by comparison, the other with resilience. Only one coding polymorphism distinguishes the two alleles, a threonine-to-serine substitution at residue 185 (186 in mouse), that is inherited in disequilibrium with multiple non-coding variants. Transcriptional studies suggest synaptic, neuronal, and cognitive preservation in human subjects with the protective haplotype, while murine *in vitro* studies reveal dramatic effects of TMEM106B deletion on neuronal development. Despite this foundation, the field has not yet resolved whether coding variant is biologically meaningful, and if so, whether it has any specific effect on neuronal phenotypes. Here we studied how loss of TMEM106B or expression of the lone coding variant in isolation affected transcriptional signatures in the mature brain and neuronal structure during development in primary neurons. Homozygous expression of the TMEM106B T186S variant in knock-in mice increased cortical expression of genes associated with excitatory synaptic function and axon outgrowth, and promoted neurite branching, dendritic spine density, and synaptic density in primary hippocampal neurons. In contrast, constitutive TMEM106B deletion affected transcriptional signatures of myelination without altering neuronal development *in vitro*. Our findings show that the T186S variant is functionally relevant and may contribute to disease resilience during neurodevelopment.

## 1. Introduction

Few genes have a significant effect on disease risk that spans multiple disorders. The endo-lysosomal transmembrane protein 106B (TMEM106B) is among this elite group, having been first been as a risk modifier of frontotemporal lobar degeneration (FTLD) (Van Deerlin et al., 2010), and subsequently as a modifier of numerous other neurodegenerative disorders including Alzheimer’s disease (AD) (Jun et al., 2016; Milind et al., 2020), hippocampal sclerosis (Rutherford et al., 2012; Murray et al., 2014; Nelson et al., 2015), and cognitive decline in amyotrophic lateral sclerosis and Parkinson’s disease (Vass et al., 2011; Tropea et al., 2019) for review see (Feng et al., 2021). While it seems intuitive to suppose that TMEM106B acts at the lysosome to promote degradation of pathological protein aggregates, several recent studies suggest an alternate mechanism of neuroprotection. Rademakers and colleagues found that cortical tissue from subjects carrying the protective TMEM106B haplotype were characterized by differential expression of genes related to synaptic transmission and retained greater levels of neuron-specific mRNAs than subjects carrying the risk allele (Ren et al., 2018). Cruchaga and co-workers similarly found preservation of neuronal signatures in subjects carrying the protective allele, which suggested a higher proportion of neurons relative to other cell types in both aged controls and cases with neurodegenerative disease (Li et al., 2020). Rhinn and Abeliovich discovered a significant association between TMEM106B genotype and transcriptional profiles of brain aging in a cohort selected specifically for the *absence* of known brain pathology. The association was replicated in a second cohort of aged individuals containing a range of neuropathologic diagnoses, where TMEM106B genotype was most influential >65 years of age (Rhinn and Abeliovich, 2017). Using the same cohort, de Jager and colleagues discovered a significant association between TMEM106B and residual cognition after accounting for the effects of age, education, gender, and ten neuropathologies common in aging (White et al., 2017). In all four studies, the protective TMEM106B allele supported signatures of brain resilience independent of pathological burden.

Manipulating TMEM106B levels *in vitro* bidirectionally alters lysosomal properties including pH, size, and cellular localization (Chen-Plotkin et al., 2012; Brady et al., 2013; Stagi et al., 2014). While lysosomes are best known for their role in protein degradation, they are also critical for neuron development, maintenance, and synaptic plasticity. Neuronal activity induces endocytosis of glutamate receptors from the postsynaptic membrane; these are either recycled to the membrane during synaptic potentiation or sorted to the lysosome upon synaptic depression (Ehlers, 2000; Martin and Henley, 2004; Fernandez-Monreal et al., 2012; Shehata et al., 2012). Lysosomes are actively transported into depolarized spines (Goo et al., 2017), where lysosomal release of cathepsin B activates extracellular proteases needed for spine enlargement during potentiation (Padamsey et al., 2017). Blocking lysosomal function decreases neurite branching and spine number, diminishes the stability of newly-enlarged spines, and lowers the frequency of spontaneous excitatory activity (Arancibia-Carcamo et al., 2009; Schwenk et al., 2014; Goo et al., 2017; Padamsey et al., 2017). During development, the endo-lysosomal system is essential for dynamic regulation of axon guidance receptors, while lysosomal enzymes are required for extracellular matrix remodeling to allow neurite outgrowth (Keleman et al., 2002; Sann et al., 2009; Ibata et al., 2019; Manzoli et al., 2021).

Given the associations between lysosome function and neuronal morphology, along with the apparent role of TMEM106B in balancing between neuroprotection and neurodegeneration, there is surprisingly little work describing how TMEM106B manipulation affects neuron development. Only one prior study has directly tested the effect of acute TMEM106B knockdown in cultured neurons to uncover marked reductions in neurite arborization, mature spine density, and synaptic protein expression (Schwenk et al., 2014). This outcome is hard to align with prior studies suggesting that the protective allele may diminish risk by *lowering* TMEM106B expression (Van Deerlin et al., 2010; Gallagher et al., 2017), which raises the possibility for an alternative mechanism of neuroprotection. While most disease-associated TMEM106B polymorphisms are in non-coding regions, these variants are in linkage disequilibrium with the lone coding polymorphism found in the protective allele (T185S in human, T186S in mouse) (Van Deerlin et al., 2010; Gallagher et al., 2017). This TMEM106B variant has been examined in only a small number of studies with mixed results (Brady et al., 2013; Nicholson et al., 2013; Jun et al., 2015; Cabron et al., 2023). Here we studied how loss of TMEM106B or expression of the lone coding variant affected transcriptional signatures in the mature brain and neuronal structure during development in primary neurons. We show that homozygous expression of the TMEM106B T186S variant in knock-in mice increased cortical expression of genes associated with synapses and neurite outgrowth, and promoted neurite branching, dendritic spine density, and synaptic density in primary hippocampal neurons. In contrast, constitutive TMEM106B deletion affected transcriptional signatures of myelination without altering neuronal development *in vitro*. Our findings show the TMEM106B T186S coding variant is functionally relevant and that its presence may support neuroprotection in aging and disease by building a stronger neuronal network during development.

## 2. Materials and methods

### 2.1. Animal generation and husbandry

The generation of TMEM106B knock-out (KO) and TMEM106B T186S knock-in (KI) animals is described in more detail in Edwards et al. (2023).

TMEM106B knock-out animals were generated by *in vitro* fertilization of C57BL/6 J oocytes using cryopreserved germplasm from Tmem106B^tm2a(KOMP)Wtsi^ mice by the BCM Genetically Engineered Mouse Core. This line was created from ES cell clone EPD0047_1_E02, generated by the Wellcome Trust Sanger Institute and made into mice by the KOMP Repository^1^ and the Mouse Biology Program^2^ at the University of California Davis. Heterozygous pups were crossed with C57BL/6 J wild-type animals (Jax strain # 000664) to maintain the line. TMEM106B^tm2a^ heterozygous animals were sequentially intercrossed with β-actin-Cre (Jax strain # 019099) and β-actin-Flp lines (Jax strain # 003800) to generate the final tm2d deletion allele. This allele lacks TMEM106B exon 4 and removes the β-galactosidase/neomycin cassette included in the tm2a starting allele. Homozygous null animals on a mixed C3B6/J genetic background were generated by mating tm2d heterozygous animals with C3HeJ wild-type females (Jax strain # 000659), and the hybrid offspring were intercrossed to produce the colony.

TMEM106B T186S knock-in animals were generated using CRISPR/Cas9 to create the T186S substitution in the endogenous mouse locus (homologous to T185S in humans). The guide RNA targeted a PAM site within exon 6 (5′-TTA CCT GCT TCA TAT CAA GT-3′). The donor DNA introduced two silent mutations along with the T to S substitution: (1) I185I (ATA to ATT) adding a novel BstBI site and (2) L191L (CTT to TTA) to disrupt the PAM site and prevent recutting of the edited genome. Melt-point analysis detected no off-target effects in 3 G1 offspring, and immunoblotting found no differences in brain TMEM106B expression between the 3 lines. Line 5825 was selected for subsequent experiments following Sanger sequencing of exon 6 to confirm the targeted mutations. Offspring were outcrossed to B6C3 (Jax strain # 100010) and maintained on a mixed C3B6/J background.

Animals were housed under a 13 h light/11 h dark cycle, with food and water provided *ad libitum*. All animal work was reviewed and approved by the Institutional Animal Care and Use Committee at Baylor College of Medicine.

### 2.2. Transcriptomic analysis

Animals between 2.5–3.5 mo of age (*n* = 6 per genotype, evenly divided between sexes) were euthanized with CO_2_, brains removed, and tissue hemisected along the midline. The right hemisphere was laid on its side to place the midline face up. After removing the olfactory bulb, the rostral aspect of the frontal cortex was isolated by a single vertical cut immediately forward of the corpus callosum. This dissection yielded a sample approximately 40–50 mg in weight that was snap frozen on dry ice and stored at −80°C until use. RNA extraction was done with the PureLink RNA Mini Kit (Life Technologies, 12183018A) paired with the PureLink DNase Set (Fisher, 12185010) to eliminate DNA contamination. RNA integrity was determined by the 260/280 ratio with a Nanodrop spectrometer and stored at −80°C until use. Samples were processed by NanoString under standard procedures (MAN-C0035-08) and mRNA detected with the mouse Neuropathology nCounter gene panel (NanoString, XT-CSO-MNROP1-12), with the addition of 30 Panel Plus custom probes targeting lysosomal, synaptic, and myelin genes. The nCounter data generated in this study has been deposited at Gene Expression Omnibus (GEO)^3^ under accession ID GSE240156.

Expression data was analyzed by NanoString using the Advanced Analysis plug-in for nSolver software (version 4.0). The dataset was first pruned using a threshold to remove genes if expression in more than 50% of samples was below the mean of negative control probes plus 2 standard deviations, calculated for each sample. This resulted in pruning of approximately 135 genes from each dataset.

Data above threshold was normalized to a subset of housekeeping genes (Aars, Asb7, Ccdc127, Cnot10, Csnk2a2, Fam104a, Lars, Mto1, Supt7l, and Tada2b) that were confirmed by GeNorm to be stable across all samples. The geometric mean of housekeeper expression in each sample was used to normalize all genes in that sample and then scaled to the average geometric mean of all samples.

Fold-change and significance were calculated using linear regression of log_2_ transformed data where each gene has a choice of two models, negative binomial or linear, based on whether the two negative binomial parameters could be estimated using a maximum likelihood method. *p* values were adjusted for multiple comparisons using the Benjamini and Yekutieli (BY) correction to account for the possibility of correlated expression between genes in the selected probe set tested here (i.e., non-independence). WT samples from the KO and KI colonies were combined to *n* = 12 for DEG analysis, based on a lack of difference between groups on the first 3 PCA axes. Comparisons with the combined WT control group were done separately for KO and KI.

Functional analysis was performed using Cytoscape (v3.9.1) and stringApp (v2.0.0). Differentially expressed genes were first identified using a cutoff of *p* < 0.05. Genes were then clustered by functional interactions using stringApp with a confidence cutoff of 0.50 and the largest initial cluster was selected for visualization. The top significant functional enrichment pathways from either GO Cellular Component or GO Biological Process were plotted in Prism (v10.0.1) using Benjamini and Hochberg (BH) false discovery rate values calculated in stringApp.

### 2.3. Viral packaging

pAAV-CBA-YFP-2A-YFP-2A-YFP (pAAV-CBA-3xYFP) was packaged in AAV8 by the Gene Vector Core at Baylor College of Medicine as described previously (Park et al., 2021). Construction of the pAAV-CBA-3xYFP plasmid is described in Kim et al. (2014).

### 2.4. Primary neuronal culture

Postnatal day 0 (P0) pups from TMEM106B heterozygous knock-out and knock-in matings were genotyped shortly after birth to identify homozygous and wild-type animals that were used for study. Pups were decapitated into cold dissociation buffer [100 mM MgCl_2_, 100 mM HEPES (Sigma, H3375), 10 mM kynurenic acid (Sigma, K3375), in Hank’s balanced salt solution (ThermoFisher, 14175095)]. Brains were removed, stripped of meninges, and the hippocampi isolated. Hippocampal dissection was performed essentially as described for late embryonic and early postnatal neuronal culture (Vicario-Abejon, 2004; Beaudoin et al., 2012; Masuch and Biber, 2019; Moutin et al., 2020; Tomassoni-Ardori et al., 2020). Hippocampal tissue was digested in papain (Fisher, 50-592-333) for 4 min at 37°C, washed with trypsin inhibitor (Sigma, T9253), and then triturated. For spine density measurements, dissociated cells were plated at a density of 150,000 cells/ml in plating media [Neurobasal-A medium (ThermoFisher, 10888022) containing B-27 (ThermoFisher, 12587010), 2 mM GlutaMAX (ThermoFisher, 35050061), and 100 U/mL pen-strep (Life Technologies, 15140122)]. Cells were grown on 12 mm coverslips that had been pre-coated with a mix of poly-D-lysine (20 μg/mL, Sigma, P6407) and laminin (3 μg/mL, Sigma, L2020). For synaptic immunostaining, cells were plated at a density of 75,000 cells/ml on 8-well slides (ibidi, 80806) that had been pre-coated with poly-D-lysine and laminin as above. Cells for both analyses were sparsely labeled with AAV8 encoding CBA-YFP-2A-YFP-2A-YFP at an MOI of 1×10^4^ shortly after plating. Cultures were maintained at 37°C, 5% CO_2_ until DIV 21. DIV 21 cultures were fixed with 4% paraformaldehyde in 1xPBS for 5 min. Coverslips used for spine and Sholl analysis were mounted without further processing onto microscope slides using Prolong Gold media (Life Technologies, P36930). All 4 genotypes were cultured and analyzed together as neonates became available from each breeding colony.

### 2.5. Immunofluorescent staining

After fixation at DIV21, cells used for synaptic immunostaining were incubated in blocking buffer [0.1% saponin (Fisher, AAA1882014), 1% BSA (Fisher, 820451) and 2% serum (Sigma, S30-M and EMD Millipore, S26)] for 1 h at RT, then incubated in primary antibody diluted in blocking solution [Bassoon (1:100, Enzo, ADI-VAM-PS003-D) and SynGAP (1:250, Cell Signaling, 5539S)] overnight at 4°C. Cells were washed in PBS then incubated in secondary antibody diluted in block solution for 1 h at RT [Gt anti-Ms IgG 568 (1:500, Molecular Probes, A11004) and Dk anti-Rb IgG 647 (1:500, ThermoFisher, A32795)]. Cells were again washed in PBS and preserved on-slide with Prolong Diamond (ThermoFisher, P36970).

### 2.6. Fluorescent imaging and analysis

For spine and synapse analysis, secondary dendrites were imaged on a Zeiss LSM 880 using AiryscanFAST mode at 63x, with 4x averaging, using z-stacks that spanned the complete z-depth. For Sholl analysis, 40x tiled images were taken on a Zeiss Axio Imager Z1 microscope. All images within an experiment were taken at a fixed exposure time and under the same laser intensity or lamp setting.

Spine density and Sholl analyses were performed using Neurolucida 360 (version 2018.2.1). For spine density analysis, images were set to 0.8 gamma, 5 black point, and 100 white point. Dendrites were reconstructed using the user-guided trace mode in the Tree module. Spine detection was done with the Spine module (outer range = 3 μm; minimum height = 0.3 μm; detector sensitivity = 100%; minimum count = 10 voxels). Spine morphology was automatically classified with Neurolucida 360. Six to nine dendrites per animal were analyzed over an average distance of 51.5 μm (range 28–97 μm, median = 52 μm KO/WT; 50 μm KI/WT) from 5 animals per genotype. For Sholl analysis, images were set to 1.0 gamma, 0 black point, and 100 white point. Cell body detection was done using the Soma module. Neurite reconstruction was done with the user-guided trace mode in the Tree module. Neurite crossings were quantified using Neurolucida Explorer at 30 μm intervals. Nine to ten neurons per animal and 5 animals per genotype were analyzed.

For synaptic marker analysis, immunolabeled pre-, post-, and co-localized puncta were analyzed with Imaris (version 9.9.0). Puncta reconstruction for bassoon was done using the Spot module with an estimated diameter of 0.6 μm and filtered for quality above 1,827. SynGAP puncta were analyzed similarly, but quality was set to >3,298. Dendrite reconstruction was done using the LabKit extension within the Surface module. Co-localized puncta were restricted to <0.50 μm from a reconstructed dendrite and < 0.50 μm from each other. Five to seven dendrites per animal were analyzed, over an average distance of 50 μm (range 25–120 μm, median = 44 μm KO/WT, 47 μm KI/WT), from 5–6 animals per genotype.

Data for each experiment was analyzed in a single batch once imaging was completed; analyses were done blind to genotype.

### 2.7. Statistical analysis

Two-tailed unpaired *t*-tests were used for spine density and synaptic puncta comparisons. Two-way mixed-effect analysis with Bonferroni post-test was used for spine morphology analysis. Two-way ANOVA with Bonferroni post-hoc test was used for Sholl analysis. All statistical tests were done using Prism 9 (version 9.2.0).

## 3. Results

### 3.1. The TMEM106B T186S variant alters transcription of synapse- and neurite-related genes in the mouse brain

**Figure 1:**
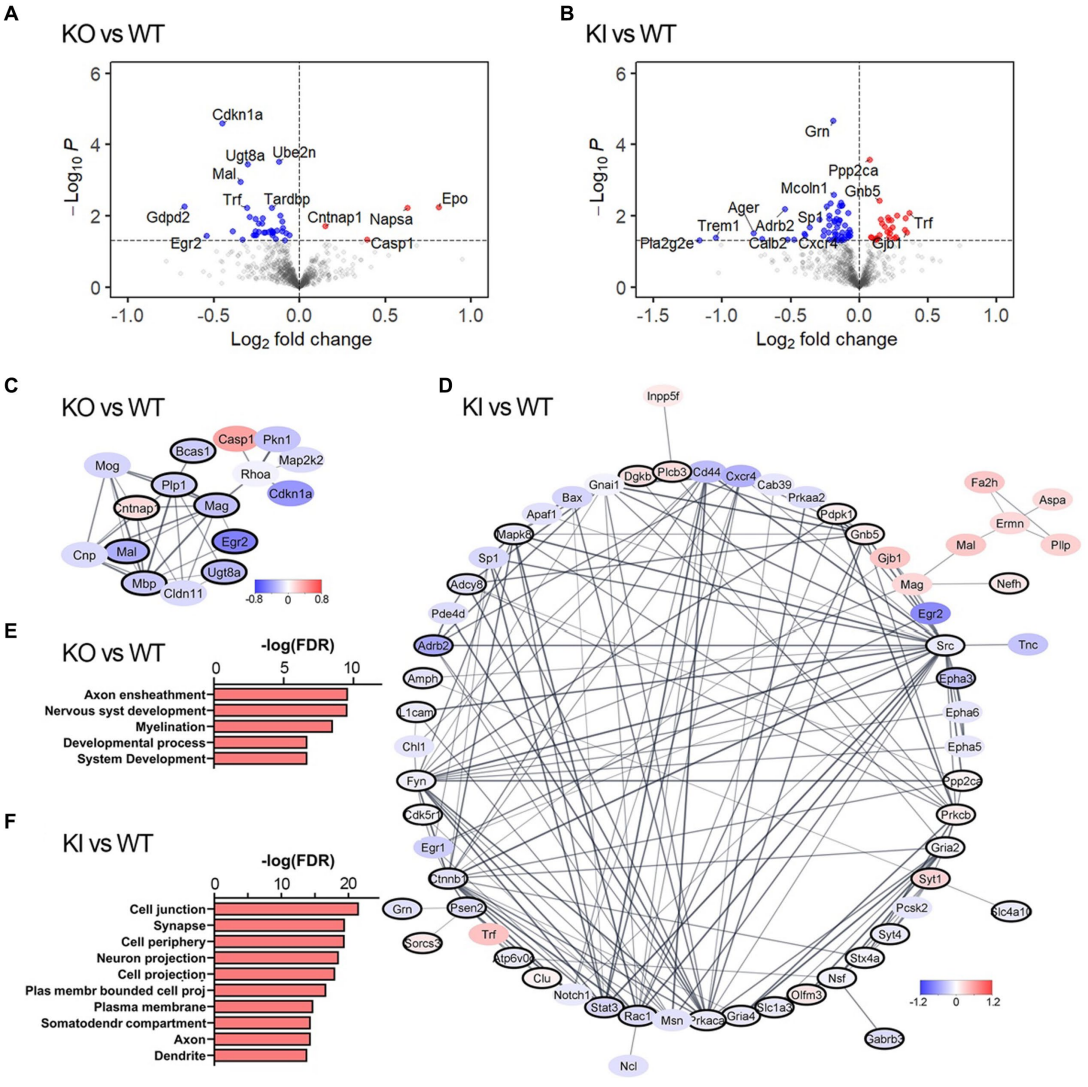
TMEM106B T186S increases transcriptional signatures of neuronal outgrowth and synapse-associated genes. **(A,B)** Volcano plot showing relative gene expression in TMEM106B KO forebrain tissue compared to WT **(A)** and TMEM106B T186S KI compared to WT **(B)**. DEGs at an unadjusted *p* < 0.05 are colored. A subset of the most changed DEGs is labeled. **(C)** The largest protein interaction network associated with transcriptional differences in KO forebrain was enriched for genes from myelin-related biological processes (black outline). Node fill color reflects log fold change relative to WT. **(D)** The predominant protein interaction network of DEGs from KI brain was enriched for synapse-related genes (black outline). All genes shown in **(C)** and **(D)** are DEGs at an unadjusted *p* < 0.05. **(E)** Top functional enrichment pathways from the GO: biological processes ontology corroborated the myelin-and development-related consequences of TMEM106B deletion. **(F)** Pathway enrichment from the GO: cellular component ontology identified synapse-and neurite-related changes in T186S brain. *n* = 6/genotype.

We performed transcriptomic analysis of brain tissue from young adult TMEM106B knock-out (KO) mice, TMEM106B T186S homozygote knock-in (KI) mice, and their respective wild-type siblings, using a NanoString neuropathology panel with 30 additional Panel Plus probes designed to supplement synaptic, lysosomal, and myelin markers. We focused our analysis on frontal cortex since this area is affected in both FTLD and AD and TMEM106B serves as a risk modifier of both. Of 789 genes detected by this platform, 42 were differentially expressed (DEGs) in KO compared to WT at an unadjusted *p*-value <0.05 (Figure 1A); 84 DEGs were identified in KI vs. WT (Figure 1B; Supplementary Table S1). Fold-change differences between genotypes were modest, and primary component analyses did not cleanly segregate genotypes by expression. Only 7 genes overlapped between the two datasets, suggesting that KO and KI affect distinct pathways in the brain rather than altering similar pathways in opposite directions. We next asked whether these DEGs corresponded to specific functional modules that might inform further phenotypic investigation. We used STRINGdb to build protein interaction networks from the DEG lists and perform functional enrichment on the resulting clusters. In KO animals few genes fell into multi-node clusters with the exception of genes related to myelination and axon ensheathment, consistent with past reports of similar TMEM106B KO models (Figures 1C,E) (Feng et al., 2020; Werner et al., 2020; Zhou et al., 2020). In contrast to the limited effect of TMEM106B deletion, KI animals showed more significant transcriptional dysregulation. A large fraction of genes altered by the T186S variant clustered within an interaction network associated with neuronal structures including synapse, neuronal projection, axon, and dendrite. Counterintuitively, many of these neurite outgrowth and synaptogenic genes were downregulated in KI relative to WT (Figures 1D,F). Downregulation of genes such as Rac1 and solute carriers such as Slc1a3 might diminish synapse formation and function, however, downregulation of genes such as EphA3, 5, and 6, Fyn, L1cam, or Notch1 might as easily result in neurite extension and synapse plasticity (Dahlhaus et al., 2008; Kania and Klein, 2016; Costa et al., 2020; Duncan et al., 2021).

### 3.2. TMEM106B T186S promotes neurite outgrowth and spine formation *in vitro*

Transcriptional profiles from adult cortex suggested that the T186S variant augmented molecular signatures of neuronal development. In contrast, signatures from KO tissue pointed to effects on myelination via changes in oligodendrocyte function. That each TMEM106B manipulation could evoke cell-type specific changes was an intriguing possibility that we examined more closely using a simplified *in vitro* system. Because the effect of TMEM106B deletion on myelin development and regeneration has been well documented in mice and human tissue (Feng et al., 2020; Zhou et al., 2020; Feng et al., 2022; Zhang et al., 2023), we decided to concentrate on neuronal development. We generated primary neuronal cultures from KO, KI, and sibling WT mice to measure synapse formation and neurite outgrowth in each genotype. Hippocampal neurons were isolated from neonatal brain within a day after birth, sparsely labeled with YFP using an AAV vector, and analyzed after 21 days *in vitro*.

**Figure 2:**
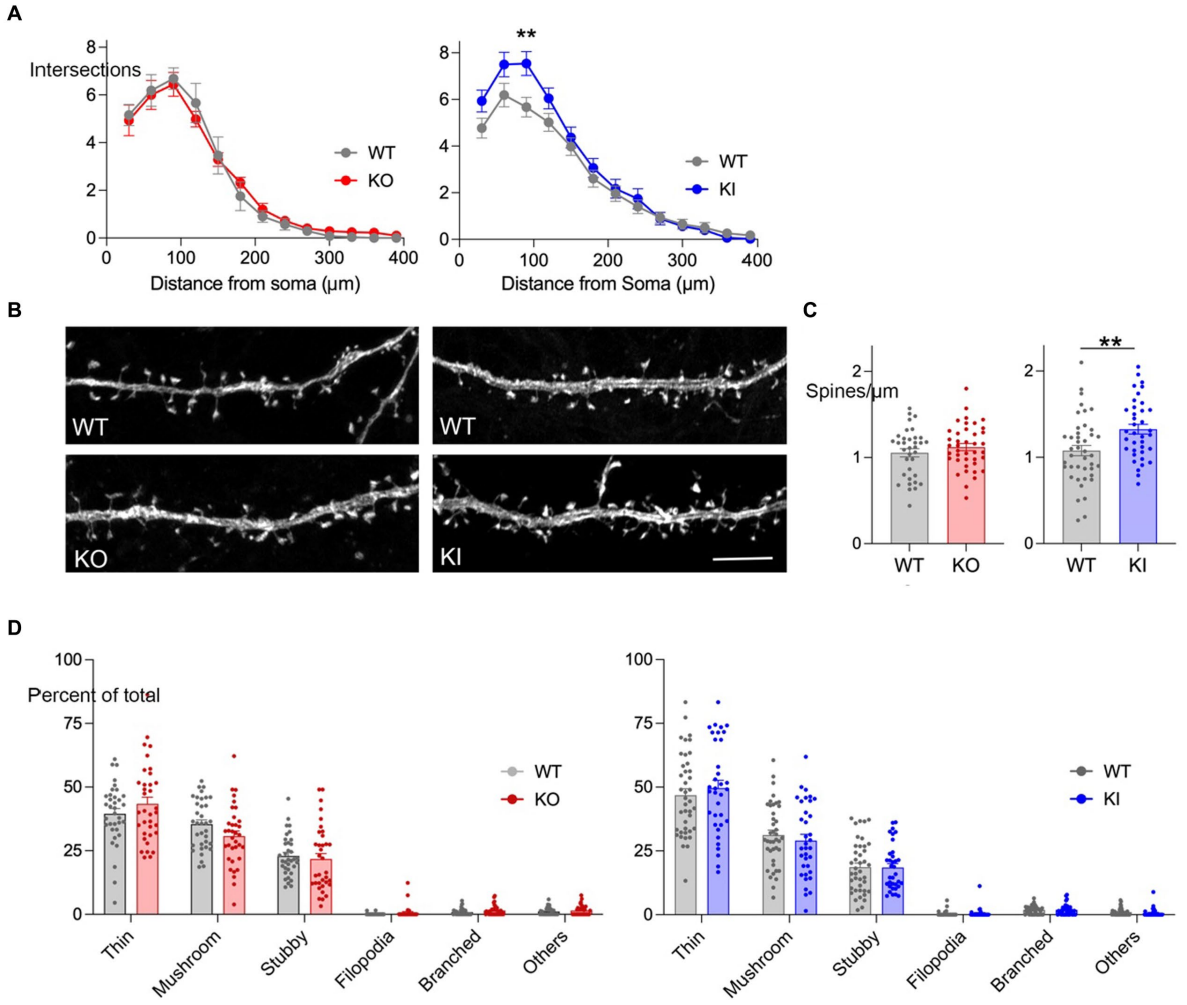
TMEM106B T186S increases neurite branching and spine density. **(A)** Sholl analysis was performed at DIV 21 on YFP-labeled hippocampal neurons from KO (left, red) and KI mice (right, blue). Graph shows the number of neurite crossings at 30 μm intervals for each genotype. *n* = 47 WT neurons and 45 KO; 48 WT and 46 KI, 5 mice/genotype. Two-way ANOVA with Bonferroni post-hoc: main effect of genotype for KO vs. WT n.s., KI vs. WT *p* < 0.0001. **(B)** Representative images of YFP-labeled secondary dendrites from each genotype. Scale bar = 5 μm. **(C)** Spine density per μm was measured for neurons from KO (left, red) and KI cultures (right, blue). Each point represents the measurement from one dendritic segment. *n* = 35 WT dendrite segments and 40 KO; 42 WT and 38 KI, 5 mice/genotype. Two-tailed unpaired *t*-test. **(D)** Spine morphology was classified into 6 categories for each dendritic segment and graphed as a percent of total (KO, left, red; KI, right, blue). Statistical comparisons were done using two-way mixed effect analysis but found no significant differences between genotypes for either model. No main effect differences were identified between the WT groups, however, post-testing prompted by an interaction between genotype and shape identified a significant difference in the percent of thin spines between control groups. **p < 0.01.

We used Sholl analysis to measure neurite arborization in cultures from each genotype. Neurons from KI mice had more neurite crossings overall than WT and the difference reached significance at 90 μm from the soma (Figure 2A). In contrast, we saw no difference in neurite crossings between KO and WT neurons.

The sparse YFP labeling also allowed us to study spine density and structure in these cultures. We focused on the initial segment of secondary dendrites, identified as the first branch away from the soma. Here again we found a significant effect of T186S which had roughly 33% more spines per μm than littermate controls (Figures 2B,C). We then studied spine morphology, classifying each into six structures (thin, mushroom, stubby, filopodia, branched, or other). Thin and mushroom spines were most common followed by stubby, with only a minor contribution of filopodia, branched or other morphologies. Despite the significant difference in T186S spine density, there was no change in the relative proportion of spine shapes compared with WT (Figure 2D). In contrast to the morphological effects of T186S expression, TMEM106B deletion had no effect on neurite branching, length, spine density, or the relative proportion of spine shapes (Figures 2A–D).

### 3.3. Increase spine density is accompanied by increased synapse density in T186S neurons

We next used immunofluorescence for pre-and post-synaptic protein markers to determine whether increased spine density seen in the KI cultures translated to an increased number of bipartite synapses. We generated new DIV 21 hippocampal neuronal cultures from neonatal KO, KI, and WT sibling controls and immunostained the fixed cells for the scaffolding protein bassoon as a marker of pre-synaptic puncta and the Ras-GTPase synGAP as a marker of post-synaptic structures. Bipartite synapses were considered when the two markers occurred less than 0.5 μm apart.

**Figure 3:**
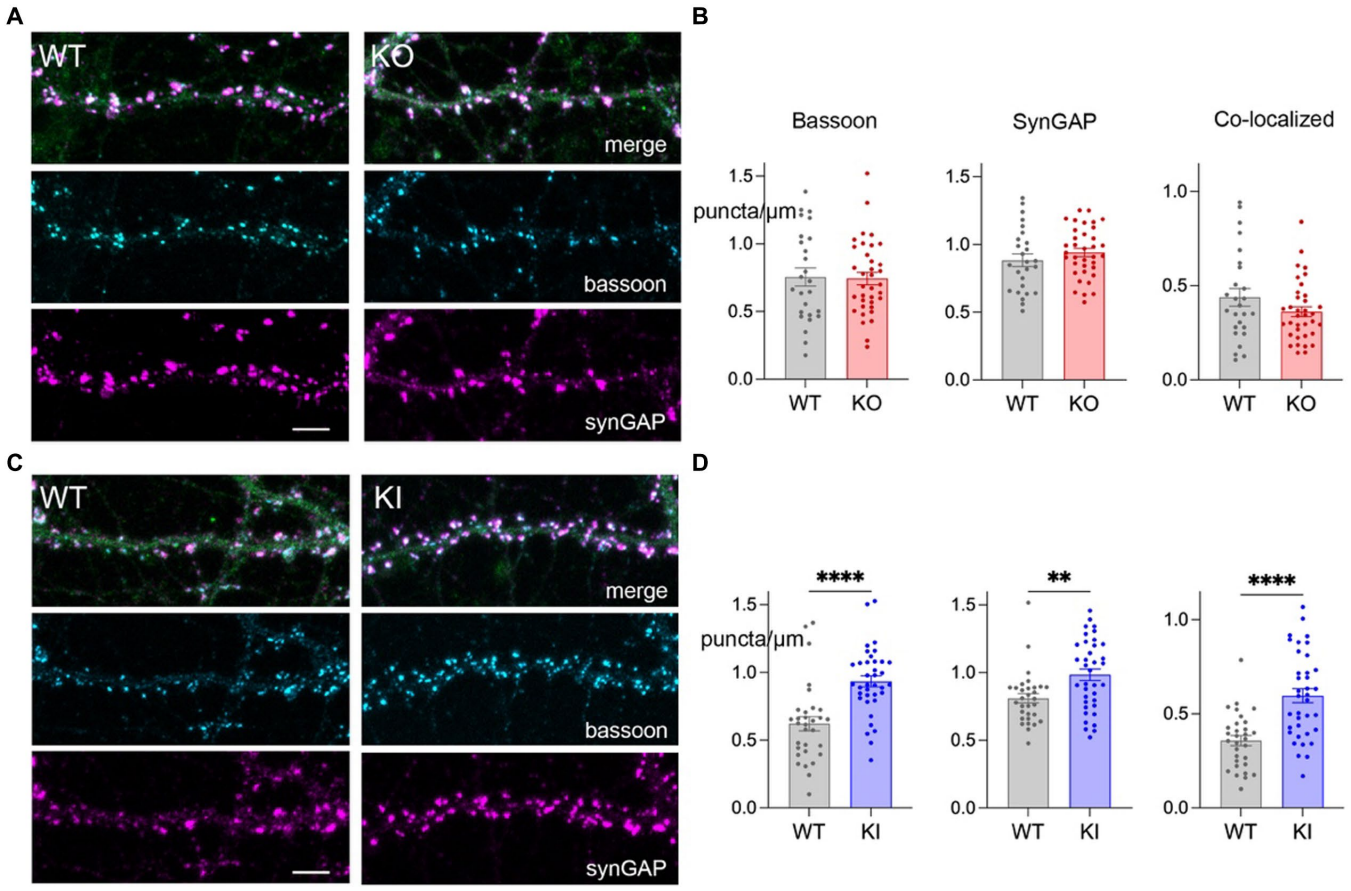
TMEM106B T186S variant increases synaptic puncta. **(A)** Representative images of secondary dendrites from primary hippocampal KO and WT neurons immunostained for the pre-synaptic marker bassoon (cyan) post-synaptic marker synGAP (magenta), and YFP (green, merge). Scale bar = 5 μm. **(B)** Linear density measurements for bassoon, synGAP, and co-localized puncta from KO and WT neurons. Each point represents the measurement of one dendritic segment. *n* = 26 WT and 36 KO, 5–6 mice/genotype. **(C)** Immunostaining of synaptic markers for KI and WT neurons, labeled as in **(A)**. **(D)** Puncta density measurements for KI and WT cultures. 31 WT and 37 KI dendritic segments, 6 mice/genotype. Two-tailed unpaired *t*-test. **p < 0.01, ****p < 0.0001.

Consistent with the increase in spine density, hippocampal neurons from KI mice had more bassoon, synGAP, and co-localized puncta per μm compared to WT controls (Figures 3C,D). In contrast, TMEM106B deletion did not affect the density of pre-or post-synaptic markers, nor that of co-localized puncta compared to WT (Figures 3A,B). These findings support our earlier discovery that the T186S variant promotes synaptic development, while loss of TMEM106B has little effect on this measure.

## 4. Discussion

Here we show that the common coding variant in TMEM106B is biologically relevant and affects neuronal phenotypes *in vitro* and *in vivo*. Our work reveals the enrichment of transcriptional signatures reflective of synaptic function and axon outgrowth in the mature brain, coupled with increased synaptic density and dendritic branching in primary neuronal cultures.

These findings build on earlier work showing that TMEM106B contains multiple non-coding SNPs which are inherited in linkage disequilibrium with a single coding variant that distinguishes the two haplotypes. Several key studies of Alzheimer’s and other dementias have uncovered transcriptional signatures of synaptic and neuronal enrichment in brain tissue from subjects carrying the T185S haplotype (Ren et al., 2018; Li et al., 2020), while in other work, presence of this allele was linked to cognitive preservation in subjects harboring neuropathology consistent with dementia (White et al., 2017). These studies suggested that TMEM106B genotype may influence dementia risk through neuronal or cognitive protection, however, co-inheritance of coding and non-coding SNPs in a single LD block prevented attribution to the T185S variant (Van Deerlin et al., 2010; Gallagher et al., 2017). Here we overcame this confound by genetically manipulating TMEM106B in mice to study the coding variant in isolation. Our findings suggest that cognitive resilience associated with the protective haplotype may in part be attributed to this single amino acid substitution and its ability to fortify neuronal structure.

These past human transcriptional studies of the TMEM106B haplotype combined with our own profiling in T186S mice lead us to examine neuronal development in more detail. Prior work from the Edbauer group offered precedent for TMEM106B manipulation to affect neuronal structure (Schwenk et al., 2014). Their studies showed that transient TMEM106B reduction in cultured rat neurons produced longer less complex dendrites, less mature spines, and lower synaptic protein levels. Given these pronounced effects of transient knockdown, we were surprised to find no change in neuronal morphology upon constitutive TMEM106B deletion. We suspect the divergent outcomes are due to methodological differences between our studies, including depth, duration, and density of TMEM106B depletion in each setting. The lack of neuronal phenotype following constitutive deletion raises the possibility of compensation by other TMEM106B homologs 106A and C, although we have no evidence for upregulation of A or C at the transcriptional level in KO mice (data not shown). An alternative explanation comes from neurodevelopmental studies reporting similar phenotypic differences between sparse knockdown and global knockout. In these settings, the divergent outcomes suggest that competition between cells with and without the targeted protein may contribute to the observed morphological effects. For example, sparse deletion of the glutamate receptor delta 2 in developing Purkinje neurons reduced overall dendritic branching, while global knock-out did not (Takeo et al., 2021). Similarly, sparse knock-down of neuroligin-1 reduced mEPSC frequency, spine and synaptic density in developing layer 2/3 pyramidal neurons, while global knock-out did not (Kwon et al., 2012). Thus, Schwenk et al. (2014) may have uncovered an important effect of TMEM106B that is only observed when inter-cellular differences in expression level exist.

Our findings raise two additional questions that we cannot yet answer. First, how does T186S influence neuronal morphology? Restated, what is the mechanism of its effect? Because TMEM106B function is still unclear, it is difficult to address how a single conservative amino acid substitution might affect its role. The T185S variant is adjacent to a glycosylation site at N183 (N184 mouse), within a consensus sequence for N-linked glycosylation (N-X-S/T). Eliminating the N183 site by mutagenesis has a dramatic impact on TMEM106B localization within the cell (Lang et al., 2012), but no one has yet examined how modifying T to S at 185 affects either glycosylation or TMEM106B localization. Based on past work showing the importance of TMEM106B in lysosomal trafficking within the neuron (Schwenk et al., 2014; Stagi et al., 2014), we hypothesized the coding variant might enhance lysosomal transit to promote structural complexity, although it is hard to envision how a luminal residue would affect cytosolic interactions required to transport lysosomes within the cell.

The second question we pose for future studies is whether the structural changes observed *in vitro* persist in the mature brain. By choosing to work in neuronal cultures, we were able to examine neuronal structure at high resolution in individual cells. Our *in vitro* findings now justify more challenging measures of morphology in the mature brain, such as might be achieved with sparse labeling and expansion microscopy. Overall, our studies demonstrate that TMEM106B T186S enhances neurite complexity and synapse density in cultured hippocampal neurons. This effect is consistent with numerous reports of neuroprotection against dementia and aging in subjects carrying this haplotype, and suggests that this variant may support denser networks capable of sustaining greater damage before succumbing to disease.

## Data availability statement

The RNA profiling dataset generated for this study can be found in the Gene Expression Omnibus (GEO) (http://www.ncbi.nlm.nih.gov/geo/) under accession number GSE240156.

## Ethics statement

The rodent studies conducted in this work were reviewed and approved by the Baylor College of Medicine Institutional Animal Care and Use Committee.

## Author contributions

QN: Visualization, Writing – original draft, Data curation, Formal analysis, Investigation. CW: Formal analysis, Investigation, Visualization, Conceptualization, Funding acquisition, Writing – review & editing. PK: Investigation, Writing – review & editing. JJ: Conceptualization, Funding acquisition, Project administration, Resources, Supervision, Visualization, Writing – original draft, Writing – review & editing.

## Footnotes

^1^ www.komp.org

^2^ www.mousebiology.org

^3^ http://www.ncbi.nlm.nih.gov/geo/
